# 1,1’-(3-Methyl-4-phenylthieno[2,3-*b*]thiophene-2,5-diyl)diethanone as a Building Block in Heterocyclic Synthesis. Novel Synthesis of Some Pyrazole and Pyrimidine Derivatives

**DOI:** 10.3390/molecules16086502

**Published:** 2011-08-03

**Authors:** Yahia Nasser Mabkhot, Abdullah Mohammed Al-Majid, Assem Barakat, Saeed Alshahrani, Yamin Siddiqui

**Affiliations:** 1 Department of Chemistry, Faculty of Science, King Saud University, P. O. Box 2455, Riyadh 11451, Saudi Arabia; 2 Department of Chemistry, Faculty of Science, Taibah University, P.O. Box 344, Medina 41411, Saudi Arabia

**Keywords:** thienothiophene, enaminones, bis-heterocycles, intramolecular cyclization, DMF-DMA

## Abstract

A series of new bis(heterocycles) featuring thieno[2,3-*b*]thiophene rings was synthesized in a combinatorial manner. Intramolecular cyclization of enaminone derivativeswith appropriate *N*-nucleophiles afforded the target compounds. All compounds were characterized by ^1^H-, ^13^C-NMR, GCMS, IR, and UV-Vis spectrometry. These compounds represent a new class of sulfur- and nitrogen-containing heterocycles that should also be of interest as new materials.

## 1. Introduction

We have recently reported a short and expedient route to various bis(heterocycles) [1,2]. The method consists of sequential directed nucleophilic addition, side chain deprotonation, nucleophilic addition, and cyclization using a nitrogen or sulfur moiety as internal nucleophile. The importance of bis(heterocycles) can be appreciated from the numerous reports in the literature in which the chemistry and biological activities of these compounds were reviewed [3,4,5,6,7,8,9,10,11]. Thus, thienothiophene derivatives exhibit biological activities, e.g., antitumor, antiviral [12,13,14,15,16,17,18], and as antibiotic, antiglaucoma drugs, or as inhibitors of platelet aggregation [19,20,21,22,23]. Substituted thienothiophenes have been used as building blocks in the synthesis of polycondensed systems which include sulfur analogues of electronic devices, due to their many fundamental advantages over their inorganic counterparts in achieving low-cost, large area and mechanically flexible electronics [24,25,26,27]. More recently thienothiophenes, in particular the the thieno[2,3-*b*]thiophene scaffold, have attracted considerable attention as the moieties offer some significant advantages such as centrosymmetry and higher rigidity, in the design of novel NLO systems by incorporating this nucleus with in unsymmetrically functionalized cyclophane [13]. On the other hand, imidazopyrimidine and triazolopyrimidine compounds have been shown antimicrobial effects [28,29,30,31,32]. Our research has been devoted to the development of new classes of bis(heterocycle) systems which incorporate the theinothiophene moiety in the hope that they may be biologically active. We report herein the use of enaminone **3** for the synthesis of new pyrimidine, pyrazole, triazolopyrimidine and imidazopyrimidine derivatives.

## 2. Results and Discussion

**Scheme 1:**
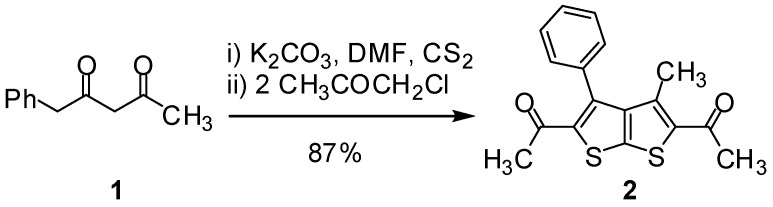
Synthesis of 1,1'-(3-methyl-4-phenylthieno[2,3-*b*] thiophene-2,5-diyl)diethanone (**2**).

The starting materials used in the synthesis, for instance, benzolyacetone (**1**), dimethylformamide dimethylacetal (DMF-DMA), 4-amino-1,2,4-triazole, and 2-aminobenzimidaole were easily available. The previously unreported 1,1'-(3-methyl-4-phenylthieno[2,3-*b*] thiophene-2,5-diyl)diethanone (**2**) was synthesized starting from benzoylacetone. Deprotonation of the active methylene proton of the latter using a K_2_CO_3_ in DMF, then trapping with a C-nucleophile such as CS_2_ and finally reaction with chloroacetone afforded the product **2** (Scheme 1). The formation of the latter was confirmed by its spectral data (IR, MS, NMR) and elemental analysis. For example, the ^1^H-NMR (DMSO-*d*_6_) spectra revealed two singlets signal at δ 1.84, 1.96 assignable to CH_3_ protons beside multiplet signal at δ 7.55 ppm assignable for 5H of the benzene ring.

**Scheme 2:**
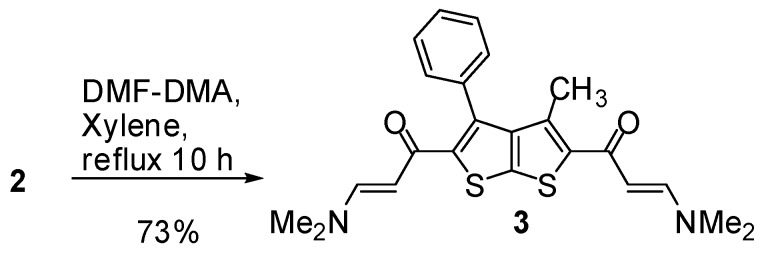
Synthesis of enaminone derivative **3**.

Condensation of **2** with dimethylformamide dimethyle acetal (DMF-DMA) under reflux for 10 h in the presence of xylene furnished enaminone **3** (Scheme 2). The ^1^H-NMR (DMSO-*d*_6_) spectrum of **3** displayed a singlet signal at δ 2.99 pmm due to the *N*,*N*-dimethyl group and a singlet at δ 1.96 ppm due to methyl protons. Two doublets signal at δ 4.53, 5.38 ppm with coupling constant *J* = 12 Hz were assignable to olefinic protons (CH=CH) in a *trans* configuration.

**Scheme 3:**
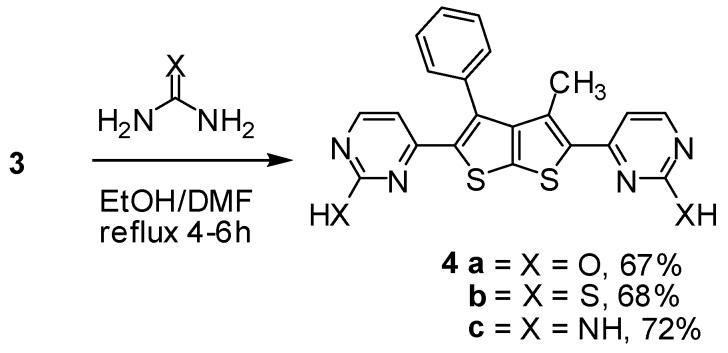
Synthesis of bis-pyrimidine derivatives **4a–c**.

**Scheme 4:**
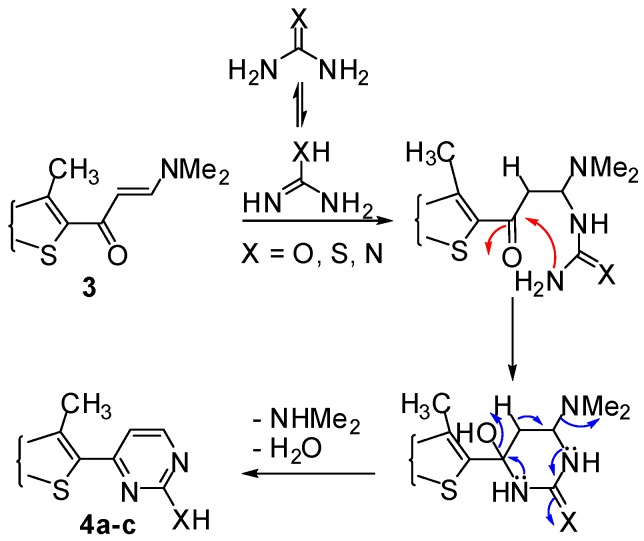
Plausible mechanism of bis-pyrimidine derivatives **4a–c**.

Reaction of enaminone **3** with an *N*-nucleophile such as urea in dioxane or EtOH/DMF mixture under reflux for 4–6 h in the presence of a catalytic amount of ZnCl_2_ as a Lewis acid afforded **4a** (Scheme 3). The structure of the product was confirmed by the ^1^H-NMR (DMSO-*d*_6_) spectrum which displayed a new pair of doublets signals at δ 5.38, 7.65 with *J* = 7.8 Hz corresponding to pyrimidinol besides a singlet peak at δ 6.5 ppm assignable to OH. The formation of compound **4a** would involve an initial addition of the amino group in urea to the activated double bond in enaminone derivative **3**, followed by deamination to an intermediate which then undergoes cyclization and aromatization *via* loss of water affording the final isolable product (Scheme 4).

Next, reaction of enaminone **3** with thiourea in refluxing DMF/Ethanol mixture afforded the corresponding dipyrimidin-2-thiol derivatives **4b** (Scheme 3). The ^1^H-NMR spectrum (DMSO-*d*_6_) of compound **4b** revealed two doublets at δ 5.36 ppm (1H, d) and 7.62 ppm (1H, d) which were readily assigned to the hydrogen attached at C_5_ and C_4_ of the pyrimidine ring respectively, and a singlet at δ 6.5 pmm (1H, s) assigned to the –SH attached at C_2_ of the pyrimidine ring. Nevertheless, when enaminone derivative **3** was reacting with guanidine under similar reaction conditions to give dipyrimidin-2-amine derivative **4c** (Scheme 3). The structure of dipyrimidin-2-amine derivatives was established on the basis of their elemental analysis and spectral data (see Experimental).

**Scheme 5:**
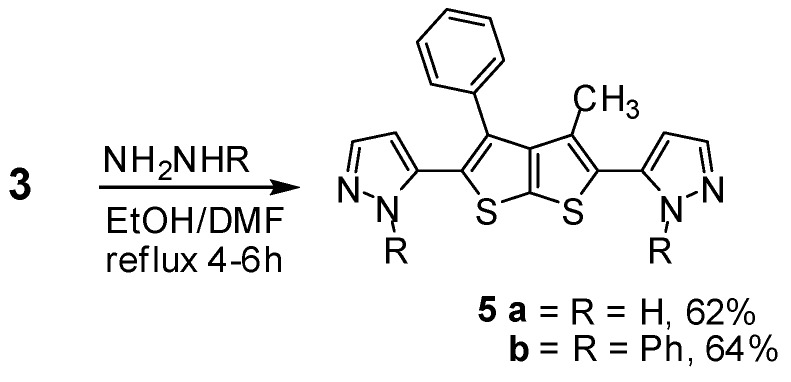
Synthesis of bis-pyrazole derivatives **5a**,**b**.

Similarly, enaminone derivative **3** cyclized with hydrazine compounds in refluxing absolute ethanol for 6 h. The novel bispyrazole **5a** was assumed to be formed via addition of the amino group in the hydrazine to the activated double bond of the enamine derivative, followed by deamination, dehydration and subsequently nucleophilic cyclization to afford the final product (Scheme 5). The IR spectrum of compound **5a** indicated the characteristic absorption band at 3402 cm^−1^ for the NH group. In particular, the ^1^H-NMR spectra (DMSO-*d*_6_) of compound **5a** revealed two doublets at δ 6.45 (1H, d) and 7.81 ppm (1H, d) which were readily assigned to the hydrogen attached at C_4_ and C_3_ of the pyrazole ring respectively, and a singlet at δ 13.01 ppm (1H, s) assigned to the –NH attached at C_2_ of the pyrazole ring. Nevertheless, when enaminone derivative **3** was reacted with phenyl hydrazine under similar reaction conditions it gave *N*-phenyl pyrazole derivative **5b** (Scheme 5). The structure of latter compound was established on the basis of their elemental analysis and spectral data (see Experimental).

**Scheme 6:**
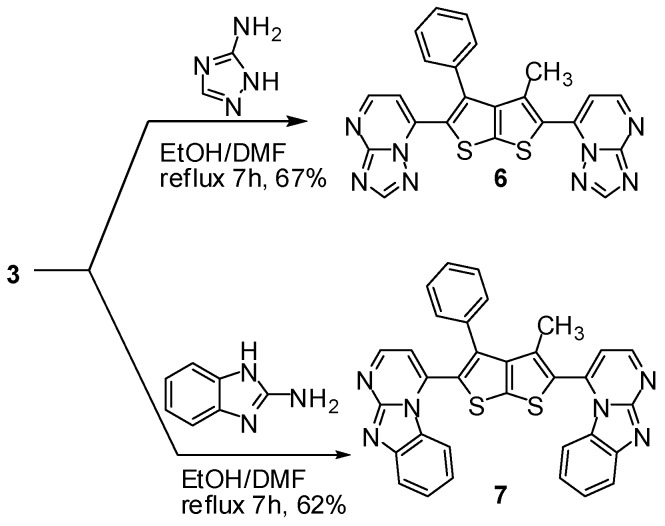
Synthesis of annelated heterocycles **6**,**7**.

The utility of enaminone **3** in the synthesis of annelated heterocycles was further explored *via* its reaction with 4-amino-1,2,4-triazole in absolute ethanol under reflux for 7 h in the presence of a catalytic amount of ZnCl_2_. It is assumed that the product **6** was formed via initial formation of a nonisolable hydrazonal followed by elimination of water to give the desired product **6** (Scheme 6). Spectral data (IR, NMR, MS) and elemental analysis were consistent with isolated product **6**. For example, the ^1^H-NMR (DMSO-*d_6_*) displayed a characteristic a pair of doublets at δ 8.17, 8.99 ppm assigned to the hydrogen at C_5_, and C_4_ of the pyrimidine ring respectively and a singlet at δ 8.67 ppm assigned to the triazole proton, respectively.

The study was extended to investigate the behavior of enaminone deriviatives **3** with different nucleophiles like 2-aminobenzimidazole with a view to synthesizing various heterocyclic ring systems. Thus, the reaction of **3** with this compound in refluxing ethanol in the presence of catalytic amount of ZnCl_2_ furnished the corresponding product **7** (Scheme 6). The structure of the product was confirmed on the basis of its elemental analysis and spectral data. The ^1^H-NMR (DMSO-*d*_6_) spectrum of the compound **7** revealed two doublets signal at δ 8.16, 8.80 assigned to the hydrogen at C_5_, and C_4_ of the pyrimidine ring, respectively.

## 3. Experimental

### 3.1. General

All melting points were measured on a Gallenkamp melting point apparatus in open glass capillaries and are uncorrected. IR spectra were measured as KBr pellets on a Perkin Elmer FT 1000 spectrophotometer. The NMR spectra were recorded on a Varian Mercury Jeol-400 NMR spectrometer. ^1^H-NMR (400 MHz) and ^13^C-NMR (100 MHz) were run in deuterated dimethylsulphoxide (DMSO-*d*_6_). Chemical shifts (δ) are referred in terms of ppm and *J*-coupling constants are given in Hz. Abbreviations for multiplicities are as follows: s (singlet), d (doublet), t (triplet), q (quartet), m (multiplet). Mass spectra were recorded on a Shimadzu GCMS-QP 1000 EX mass spectrometer at 70 eV. Elemental analysis was carried out on an Elementar Vario EL analyzer.

### 3.2. 1,1'-(3-Methyl-4-phenylthieno[2,3-b]thiophene-2,5-diyl)diethanone (**2**)

A mixture of benzoylacetone **1** (16.2 g, 0.1 mol) and anhydrous potassium carbonate (25 g) in DMF (30–40 mL) was stirred vigorously at room temperature for 5 min, then carbon disulfide (7.6 mL, 0.1 mol) was added with continued stirring for 30 min. The resulting reaction mixture were cooled in ice bath, then chloroacetone (18.5 mL, 0.2 mol) was added with continued stirring for 15 min, then cooling bath subsequently removed and the mixture was stirred for further 30 min. The solid product was collected by filtration and washed with water, dried and the crude product was recrystallized from glacial acetic acid to give a pale green crystals. Yield: 87%; m.p. 204–206 °C; IR (_νmax_): 1645 (C=O) cm^−1^; ^1^H-NMR δ (ppm): 1.84 (s, 6H, CH_3_), 1.96 (s, 3H, CH_3_), 7.551–7.61 (m, 5H, C_6_H_5_); ^13^C-NMR δ (ppm): 14.49 (CH_3_), 29.37–30.55 (COCH_3_), 192.2 (C=O), 129.23, 129.55, 129.87, 134.79, 138.82, 141.84, 147.68 (Ar–C); MS *m/z* (%): 314 [M+, 70%], 299 (100), 226 (37), 184 (14); Anal. calcd. for C_17_H_14_O_2_S_2_: C, 64.94; H, 4.49; S, 20.40; Found: C, 64.95; H, 4.44; S, 20.43.

### 3.3. 1,1'-(3-Methyl-4-phenylthieno[2,3-b]thiophene-2,5-diyl)bis(3-(dimethylamino)prop-2-en-1-one) (**3**)

A mixture of compound **2** (1.75 g, 5 mmol), DMF-DMA (1.19 mL, 0.01 mol) was refluxed in *m*-xylene (15 mL) for 10 h. After cooling, the resulting solid product was collected by filtration to give a dark yellow crystals. Yield: 73%; m.p. 250 °C; IR (_νmax_): 1622 (C=O) cm^−1^; ^1^H-NMR δ (ppm): 1.96 (s, 3H, CH_3_), 2.99 (s, 12H, CH_3_), 4.53 (d, 1H, *J* = 12 Hz, CH), 5.38 (d, 1H, *J* = 12 Hz, CH), 7.41–7.65 (m, 5H, C_6_H_5_); ^13^C-NMR δ (ppm): 14.9 (–CH_3_), 44.79 (–N=(CH_3_)_2_), 109.8 (–CO–CH=), 153.9 (=CH–N), 180 (C=O); MS *m/z* (%): 424 [M+, 57%], 380 (51), 336 (18), 309 (18), 98 (100); Anal. calcd. for C_23_H_24_N_2_O_2_S_2_: C, 65.06; H, 5.70; N, 6.60; S, 15.10; Found: C, 65.10; H, 5.68; S, 15.07.

### 3.4. General Procedure for the Synthesis of Compounds **4a–c** (GP1)

A mixture of compound **3** (0.212 g, 0.5 mmol), urea dervitives (2 equiv., 1 mmol) refluxed in dioxane (20 mL) for 4–6 h after in the presence of 0.5 mL of TEA and catalytic amount of ZnCl_2_. After cooling, the resulting solid products were filtered off, washed with ethanol, dried and recrystallized from DMF/EtOH, afford the corresponding derivatives **4a–c**, respectively.

*4,4'-(3-Methyl-4-phenylthieno[2,3-b]thiophene-2,5-diyl) dipyrimidin-2-ol* (**4a**). Compound **4a** was prepared from urea following GP1 as a pale yellow crystalline powder. Yield: 67%; m.p. 248 °C; IR (_νmax_): 3444 (OH), 1624 (C=N) cm^−1^; ^1^H-NMR δ (ppm): 1.96 (s, 3H, CH_3_), 5.38 (d, 1H, *J* = 7.8 Hz, CH), 6.5 (s, 1H, O–H), 7.65 (d, 1H, *J* = 7.8 Hz, CH), 7.41–7.65 (m, 5H, C_6_H_5_); MS *m/z* (%): 418 [M+, 2%]; Anal. calcd. for C_21_H_14_N_4_O_2_S_2_: C, 60.27; H, 3.37; N, 13.39; O, 7.65; S, 15.32; Found: C, 60.24; H, 3.31; N, 13.38; S, 15.32.

*4,4'-(3-Methyl-4-phenylthieno[2,3-b]thiophene-2,5-diyl) dipyrimidin-2-thiol* (**4b**). Compound **4b** was prepared from thiourea following GP1 as a pale yellow crystalline powder. Yield: 68%; m.p. 247 °C; IR (_νmax_): 1625 (C=N) cm^−1^; ^1^H-NMR δ (ppm): 1.96 (s, 3H, CH_3_), 5.36 (d, 1H, *J* = 7.8 Hz, CH), 6.5 (s, 1H, S–H), 7.41–7.65 (m, 5H, C_6_H_5_), 7.62 (d, 1H, *J* = 7.8 Hz, CH); MS *m/z* (%): 450 [M+, 2%]; Anal. calcd. for C_21_H_14_N_4_S_4_: C, 55.97; H, 3.13; N, 12.43; S, 28.46; Found: C, 55.98; H, 3.12; N, 12.41; S, 28.41.

*4,4'-(3-Methyl-4-phenylthieno[2,3-b]thiophene-2,5-diyl) dipyrimidin-2-amine* (**4c**). Compound **4c** was prepared from guanidine following GP1 as a yellow crystaline powder. Yield: 72%; m.p. 246 °C; IR (_νmax_): 3419 (NH_2_), 1624 (C=N) cm^−1^; ^1^H-NMR δ (ppm): 1.96 (s, 3H, CH_3_), 4.50–4.53 (d, 2H, NH_2_), 5.39 (d, 1H, *J* = 11.7 Hz, CH), 7.41–7.52 (m, 5H, C_6_H_5_), 7.66 (d, 1H, *J* = 11.7 Hz, CH); ^13^C-NMR δ (ppm): 14.99, 19.12, 56.58, 94.12, 108, 128, 129, 130, 136, 154, 179; MS *m/z* (%): 416 [M+, 2%], 336(100), 324(47), 153(8); Anal. calcd. for C_21_H_16_N_6_S_2_: C, 60.55; H, 3.87; N, 20.18; S, 15.40; Found: C, 60.58; H, 3.85; N, 20.15; S, 15.38.

### 3.5. General Procedure for the Synthesis of Compounds **5a–b** (GP2)

A mixture of compound **3** (1 mmol), and an excess of hydrazine derivatives (1 mL) refluxed in EtOH (20 mL) for 6 h. After cooling, the resulting solid products were filtered off, washed with ethanol, dried and recrystallized from MeOH, afforded the corresponding derivatives **5a**,**b**.

*3,3'-(3-Methyl-4-phenylthieno[2,3-b]thiophene-2,5-diyl)bis(1H-pyrazole)* (**5a**). Compound **5a** was prepared from hydrazine hydrate following GP2 as white crystals. Yield: 62%; m.p. 177 °C; IR (_νmax_): 3402 (NH), 1624 (C=N) cm^−1^; ^1^H-NMR δ (ppm): 1.87 (s, 3H, CH_3_), 6.45 (d, 1H, *J* = 4.5 Hz, CH), 7.53–7.40 (m, 5H, C_6_H_5_), 7.81 (d, 1H, *J* = 4.5 Hz, CH), 13.01 (s, 1H, NH); ^13^C-NMR δ (ppm): 14.03(CH_3_), 103(CH), 145 (N=CH), 128.62, 129.13, 129.96, 130.35, 130.54, 136.37, 147.33 (Ar–C); MS *m/z* (%): 362 [M+, 43%]; Anal. calcd. for C_19_H_14_N_4_S_2_: C, 62.96; H, 3.89; N, 15.46; S, 17.69; Found: C, 62.98; H, 3.86; N, 15.45; S, 15.72.

*3,3'-(3-Methyl-4-phenylthieno[2,3-b]thiophene-2,5-diyl)bis(1-phenyl-1H-pyrazole)* (**5b**). Compound **5b** was prepared from phenyl hydrazine following GP2 as brown crystals. Yield: 64%; m.p. 199 °C; IR (_νmax_): 1606 (C=N) cm^−1^; ^1^H-NMR δ (ppm): 1.86 (s, 3H, CH_3_), 6.48 (d, 1H, *J* = 4.5 Hz, CH), 6.53–7.20 (m, 15H, C_6_H_5_), 7.55 (d, 1H, *J* = 4.5 Hz, CH); ^13^C-NMR δ (ppm): 14.03 (CH_3_), 102 (CH), 143 (N=CH), 128.52, 129.23, 129.94, 130.38, 130.54, 136.38, 147.33, 152.84 (Ar–C); MS *m/z* (%): 514 [M+, 1%]; 169 (5), 107 (100), 92 (55), 90 (35); Anal. calcd. for C_3_1H_22_N_4_S_2_: C, 72.34; H, 4.31; N, 10.89; S, 12.46; Found: C, 72.36; H, 4.29; N, 10.86; S, 12.43.

### 3.6. General Procedure for the Synthesis of Compounds **6**,**7** (GP3)

To a solution of compound **3** (212 mg, 0.5 mmol) in DMF (2 mL), substituted amine (2 equiv., 1 mmol) in EtOH (20 mL, 99.9%) was added, then the resulting reaction mixture was heated under reflux for 7 h in the presence of catalytic amount of ZnCl_2_. After cooling, the solid product was collected by filtration, washed with ethanol, dried and recrystallized from DMF/EtOH, to afford the corresponding derivatives **6**,**7**, respectively.

*7,7'-(3-Methyl-4-phenylthieno[2,3-b]thiophene-2,5-diyl)di-[1,2,4]triazolo[1,5-a]pyrimidine* (**6**). According to GP3, **6** was obtained from 4-amino-1,2,4-triazole (84 mg) as yellow crystals. Yield: 67%; m.p. 245 °C; IR (_νmax_): 1624 (C=N) cm^−1^; ^1^H-NMR δ (ppm): 1.96 (s, 3H, CH_3_), 7.41–7.56 (m, 5H, C_6_H_5_), 8.17 (d, 1H, *J* = 8.5 Hz, CH, pyrimidyl), 8.67 (s, 1H, CH, triazole), 8.99 (d, 1H, *J* = 12.5 Hz, CH, pyrimidyl); ^13^C-NMR δ (ppm): 14.29 (–CH_3_), 115.38, 125,129.11, 129.98, 130.21, 132.81, 135.8, 152.8, 154.43, 159.98, 162.88 (Ar–C); MS *m/z* (%): 466 [M+,45%]; Anal. calcd. for C_23_H_14_N_8_S_2_: C, 59.21; H, 3.02; N, 24.02; S, 13.75; Found: C, 59.19; H, 3.03; N, 24.04; S, 13.71.

*2,2'-(3-methyl-4-phenylthieno[2,3-b]thiophene-2,5-diyl)bis(benzo[4,5]imidazo [1,2-a]pyrimidine)* (**7**). According to GP3, **7** was obtained from 2-aminobenzimidazole (133 mg) as dark yellow crystals. Yield: 62%; m.p. 245 °C; IR (_νmax_): 1622 (C=N) cm^−1^; ^1^H-NMR δ (ppm): 1.96 (s, 3H, CH_3_), 7.42–7.65 (m, 8H, C_6_H_5_, benzimidazole), 8.16 (d, 1H, *J* = 12.5Hz, CH, pyrimidyl), 8.39 (d, 1H, *J* = 8.5Hz, CH, benzimidazole), 8.80 (d, 1H, *J* = 12.5 Hz, CH, pyrimidyl); ^13^C-NMR δ (ppm): 14.81(–CH_3_), 112.05, 115.13, 122.5, 127.13, 128.1, 129.9, 131.31, 135.97, 139.1, 142.0, 148.4, 156 (Ar–C); MS *m/z* (%): 564 [M+, 1%], 488 (1.5), 380 (38), 336 (55), 324 (67), 98 (100); Anal. calcd. for C_33_H_20_N_6_S_2_: C, 70.19; H, 3.57; N, 14.88; S, 11.36; Found: C, 70.21; H, 3.54; N, 14.85; S, 11.40.

## 4. Conclusions

In conclusion, the present investigation describes an efficient method for preparing novel bis(heterocycles), many of which may display potentially interesting biological activity in the field of medicinal chemistry.
